# Interpolated pretesting can boost memory of related and distinct prose materials

**DOI:** 10.1007/s00426-024-02042-8

**Published:** 2024-11-13

**Authors:** Oliver Kliegl, Karl-Heinz T. Bäuml

**Affiliations:** Department of Experimental Psychology, Regensburg University, Regensburg, 93040 Germany

## Abstract

The pretesting effect refers to the finding that tests performed before to-be learned material is encountered can enhance later retention of the material, even when no correct answers were provided on the initial pretest. The goal of the present study was to examine whether interspersing pretest questions between the study of multiple segments consisting of prose passages can induce a pretesting effect on a final cumulative recall test on all segments. To this end, participants studied four segments which were either thematically related (Experiment 1) or distinct (Experiment 2) and either received pretest questions about each segment immediately prior to study of the segment (pretest condition) or not (study-only condition). Results of the cumulative final test performed 24 h after study of the segments showed for both experiments that interpolated pretesting enhanced correct recall of the segments. The findings thus suggest that the positive effects of pretesting on memory generalize from the standard single-list design to a multiplelists design when pretests are performed prior to study of each list. Interpolated pretesting thus may play a critical role as a potential learning tool in educational practice.

## Interpolated pretesting can boost memory of related and distinct prose

### Materials

Memory tests not only provide a tool for assessing a person’s current level of knowledge, but are themselves effective learning strategies that can boost memory performance (for reviews, see Bäuml & Kliegl, 2024; Karpicke, 2017). Remarkably, even tests given before learning can enhance recall on a later test (e.g., Kornell et al., 2009; Richland et al., 2009; for a review, see Pan & Carpenter, 2023). For instance, Richland et al. (2009) showed that having participants guess the answers to prequestions (e.g., ‘What is total color blindness caused by brain damage called?’) before they read a passage about color blindness containing the answers to the prequestions enhanced their memory performance when they again received these questions on a subsequent final test, relative to participants who studied the passage without receiving any initial questions. This pretesting effect is particularly striking since studies examining the phenomenon usually exclude correct guessing attempts made during initial pretesting from the data analysis, which means that the effects of failed guessing attempts are isolated. From an applied perspective, the pretesting effect appears highly relevant since it has been observed for various types of study materials, such as trivia questions, word pairs, videos, and prose passages, and has not only been found in laboratory-based studies, but also in educational settings (for reviews, see Chan et al., 2018; Kornell & Vaughn, 2016).

A critical issue that arises for educational contexts is whether pretesting can be used to promote the acquisition of study material that is distributed over multiple segments such as, for instance, multiple book chapters. Indeed, as students approach the exam phase, they often have to prepare large chunks of information that are either related because they belong to a single subject or largely independent of one another because tests for different subjects have to be prepared in parallel. For both types of situations, students who want to use pretesting as a learning tool may not only ask themselves prequestions about the material to be studied at the beginning of the learning period but rather throughout the whole process, e.g., prior to reading a new book chapter. Thus far, little is known about whether such interpolated pretesting can boost long-term retention and whether the effectiveness of such a technique depends on whether the segments to be studied are thematically related or distinct.

The findings from one earlier study by Pan et al. (2020) suggest that interpolated pretesting may boost later retention of related study material. These researchers conducted two experiments, in each of which participants watched a 26-minute video of a statistics online lecture that was partitioned into four segments of similar length. Before participants watched each segment, they either solved algebra problems or had to answer multiple-choice questions about the segment. Both Experiments 1 and 2 showed that interpolated pretesting led to higher recall performance and reduced mind wandering on a subsequent final test on all four segments than interpolated solving of algebra problems. The results of Experiment 2 further indicated that the benefits of such interpolated pretesting were similar to the benefits of (more typical) pretesting administered entirely prior to segment presentation. The findings thus suggest that interpolated pretesting can keep participants engaged with a learning task that consists of several related segments.

While these findings sound promising, it is important to examine their generalizability, especially since several aspects of the Pan et al. (2020) study diverge from typical pretesting-effect procedures. In particular, the researchers (i) used a topic as study material that their participants (i.e., undergraduate psychology students) may already have been familiar with to some degree (i.e., signal detection theory) and (ii) applied multiple-choice questions during the initial pretest. Both the topic and the type of initial test may have led to the relatively high percentage of correct answers on the pretest (i.e., 51% in Experiment 1 and 48% in Experiment 2). Unlike in many other pretesting-effect studies which removed questions for which correct answers were provided on the initial pretest from further analysis, the researchers also did not isolate the effects of erroneous guesses on later recall performance. While the decision not to distinguish between correctly and incorrectly answered questions on the initial pretest seems fine from a purely applied perspective, it is important to consider in the next step for a more typical version of the task that excludes all initial correct answers whether interpolated pretesting can still induce a continuous pretesting effect across all study segments.

A related issue to consider is the potential role of the success rate during initial pretesting for the effects of interpolated testing: the possibility arises that pretest questions which lead to a lower success rate than in the Pan et al. (2020) study – most studies of the pretesting effect in fact report success rates of under 10% (e.g., Grimaldi & Karpicke, 2012; Kliegl et al., 2024a; Kornell et al., 2009) – could reduce participants’ engagement over multiple study segments in the pretesting condition and thus reduce the size of the pretesting effect from earlier to later study segments. Indeed, it seems plausible that when participants realize that they answered most or all pretest questions incorrectly as soon as they read the first study segment, their effort to come up with adequate guesses on subsequent interpolated pretest cycles may diminish. The current study therefore examined whether interpolated pretesting can still boost later retention when the pretest questions are so difficult that mostly errors are produced on the initial pretest and when only questions for which errors were produced on the initial pretest are included into the further analyses.

### The present study

The goal of the present study was to examine whether pretests that are interspersed between single study segments can boost later recall performance both when the segments consist of related and when they consist of distinct prose passages. In Experiment 1, participants were shown a text about Big Bang theory that was divided into four study segments. Participants were either asked to study each segment for a later test (study-only condition) or, prior to studying each segment, to answer seven questions about the immediately following segment (pretest condition; e.g.,” How many years ago did the Big Bang set the expansion of the universe in motion?). Unlike in the Pan et al. (2020) study, no answer options were shown. Study duration in the pretest condition was one third shorter than in the study-only condition (2 min 20 s vs. 3 min 30 s) to account for the duration of the pretest, which took 1 min 10 s (for a similar proceeding, see Richland et al., 2009). Twenty-four hours after the acquisition phase, participants engaged in a final test on all four segments. This test included in random order all 28 initial pretest questions, i.e., seven questions from each of the four segments. Percentage of correctly answered final-test questions and number of overt errors (i.e., intrusions) produced on the final test were analyzed.

Experiment 1 was intended as a conceptual replication of Pan et al. (2020), examining whether their finding that interpolated pretesting can lead to an overall boost in final-test performance still arises when a more difficult free-answer format instead of a multiple-choice answer format is used. Procedural details of Experiment 2 were mostly identical to Experiment 1, but with the critical difference that the four study segments consisted of texts that were unrelated to each other. The goal of Experiment 2 thus was to investigate whether interpolated pretesting can still benefit learning and memory of multiple study segments when they belong to different topics.

## Experiment 1

### Method

#### Participants

We used previous pretesting-effect studies which manipulated type of practice (study only vs. pretest) as a between-subjects variable as a starting point for determining sample size (e.g., Pan et al., 2020; Richland et al., 2009). Using this heuristic, 72 students at Regensburg University were recruited to take part in the experiment overall (mean age = 25.3 years; 42 female, 30 male, 0 diverse), 36 of them in each of the two experimental conditions. All participants spoke German as their native language. Participants gave their spoken informed consent and received either course credit or a compensatory amount of money for their participation. Both experiments were carried out in accordance with the provisions of the World Medical Association’s Declaration of Helsinki.

#### Material

Four related text passages about Big Bang theory (adapted from Chan, 2009) were used as study material. Each passage was approximately 140 words long. The four passages were always presented in the same order, which was necessary because passages built upon each other and thus could only be understood in this particular order. All four passages were translated into German.

#### Design and procedure

Experiment 1 was conducted online via one-on-one meetings using the videoconferencing software program Zoom (Zoom Video Communications). The experiment consisted of two distinct phases: An acquisition phase and a final test phase. The critical study-format manipulation (study only vs. pretest) occurred during the initial acquisition phase. In the study-only condition, participants were shown the four prose passages during this phase for 3 min and 30 s each and were asked to study the content of each passage for a later test. In the pretest condition, participants were only shown each passage for 2 min and 20 s during the acquisition phase and completed a pretest prior to studying each passage. This pretest consisted of seven questions about the immediately following passage with each question displayed in the center of the screen for 10 s each. Participants were asked to respond orally to each question within the 10 s time frame, even if they had to guess. The experimenter recorded their responses in writing. Twenty-four hrs after the acquisition phase, participants returned for a second meeting on which they engaged in the final-test phase. The final test consisted of all 28 pretest items (i.e., the seven questions per passage), which were shown for 10 s each in a completely random order. As in the pretest, participants were asked to respond orally, with the experimenter documenting the answers in writing.

### Results of experiment 1

#### Initial pretest

On the initial pretest, participants responded correctly to 0.8% of the questions of study segment 1, 2.0% of the questions of study segment 2, 1.6% of the questions of study segment 3, and 2.4% of the questions of study segment 4. The difference in correct responses between segments was not reliable, *F*(3,105) *<* 1. Since the focus of this study was on the effects of erroneous guesses on subsequent memory, we excluded from further analyses all questions for which correct answers were made during pretesting.

#### Final test – correct recall

**Fig. 1:**
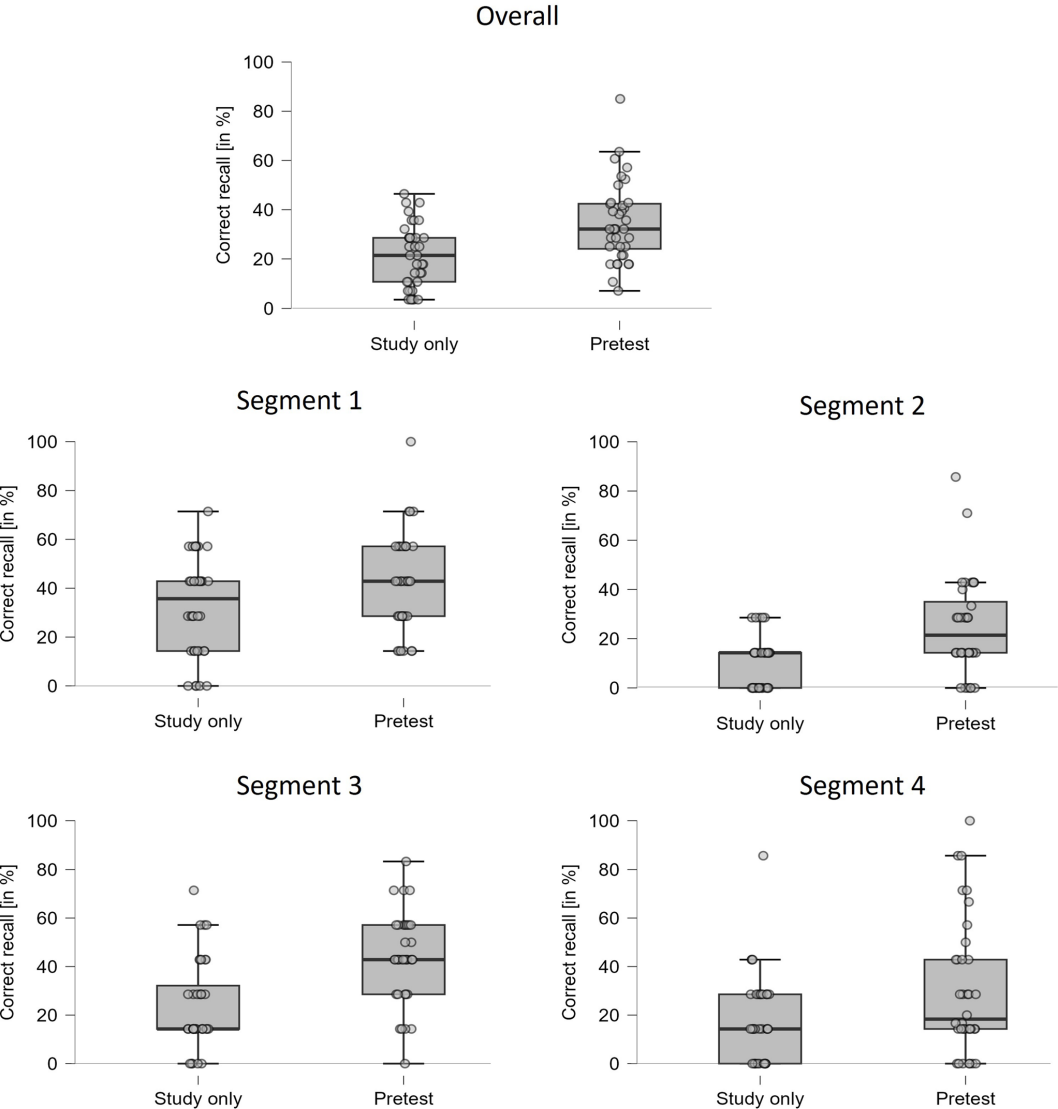
Results of Experiment 1. Box plots with jittered data points depicting recall performance on the final test (in %) as a function of TYPE OF PRACTICE (study only vs. pretest). Each data point reflects recall performance of a single participants. The box plot at the top of the figure shows recall performance averaged across all four segments. The box plots below show mean recall performance separately for the four text segments

Figure 1 shows the percentage of correctly answered questions on the final test as a function of the between-subjects factor TYPE OF PRACTICE (study only vs. pretest) and the within-subjects factor STUDY SEGMENT (segment 1 vs. segment 2 vs. segment 3 vs. segment 4). A 2 × 4 ANOVA of the two factors on correct-recall performance revealed significant main effects of type of practice, *F*(1,70) = 15.49, *MSE* = 856.30, *p <* .001, *η*_*p*_^2^ = 0.18, and study segment, *F*(3,210) = 26.09, *MSE* = 223.71, *p <* .001, *η*_*p*_^2^ = 0.27, reflecting a reliable pretesting effect with overall higher recall rates for the pretest than the study-only condition (34.9% vs. 21.3%) and reflecting that overall recall differed between study segments (37.1% vs. 17.3% vs. 33.7% vs. 24.4%). Most important, there was no interaction between the two factors, *F*(3,210) = 1.47, *MSE* = 223.7, *p* = .221, *η*_*p*_^2^ = 0.02, suggesting that the magnitude of the pretesting effect did not vary reliably across segments.

#### Final test – intrusions

All overt incorrect responses that participants produced on the final test were counted as intrusions. Table 1 shows the number of intrusions produced on the final test as a function of the factors TYPE OF PRACTICE and STUDY SEGMENT. A 2 × 4 ANOVA of the two factors on number of intrusions revealed a significant main effect of STUDY SEGMENT, *F*(3,210) = 8.65, *MSE* = 1.73, *p <* .001, *η*_*p*_^2^ = 0.11, reflecting that overall intrusion levels differed between study segments (2.6 vs. 3.2 vs. 2.5 vs. 2.1%). There was no main effect of TYPE OF PRACTICE, *F*(1,70) = 2.05, *MSE* = 2.99, *p* = .16, *η*_*p*_^2^ = 0.03, and no interaction between factors, *F*(3,210) *<* 1.

**Table 1:**
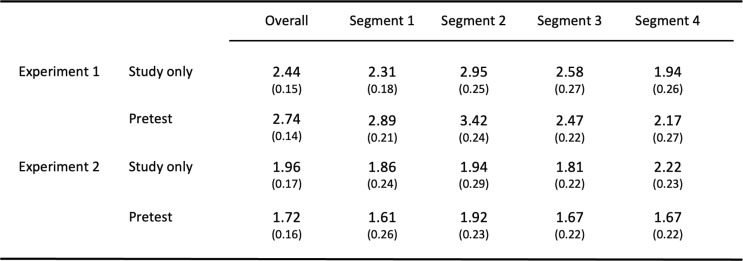
Mean number of instrusions together with standard errors of the mean(in brackets) on the final test as a function of TYPE OF PRACTIC and STUDY SEGMENT

## Experiment 2

### Method

*Participants*. Applying the same heuristic as in Experiment 1 to determine sample size, 72 participants were recruited for Experiment 2, with 36 participants in each of the two experimental conditions (mean age = 24.2 years; 45 female, 27 male, 0 diverse). All participants spoke German as their native language and gave their spoken informed consent. In return for their participation, all subject received either course credit or a compensatory amount of money.

*Material*,* design and procedure*. Experiment 2 was identical to Experiment 1, with two critical exceptions: First, instead of four related prose passages, we used four prose passages as study material that covered different topics and were thus unrelated to each other. Each passage was approximately 140 words in length and was already used in prior studies, covering ‘neanderthals’ (Fritz & Morris, 2015), ‘porcupines’ (Divis & Benjamin, 2014), ‘chronic wasting disease’ (Divis & Benjamin, 2014), and ‘uses of garlic’ (Fritz & Morris, 2015). All four prose passages were translated into German. Second, presentation order of the passages was balanced, meaning that across participants, each passage served equally often as the first study segment, the second study segment, the third study segment, and fourth study segment.

### Results of experiment 2

#### Initial pretest

On the initial pretest, participants responded correctly to 4.4% of the questions of study segment 1, 5.6% of the questions of study segment 2, 7.5% of the questions of study segment 3, and 7.1% of the questions of study segment 4. The difference in correct responses between segments was not reliable, *F*(3,105) *<* 1. Since the focus of this study was on the effects of erroneous guesses on subsequent memory, we excluded from further analyses all questions for which correct answers were made during pretesting.

#### Final test – correct recall

**Fig. 2:**
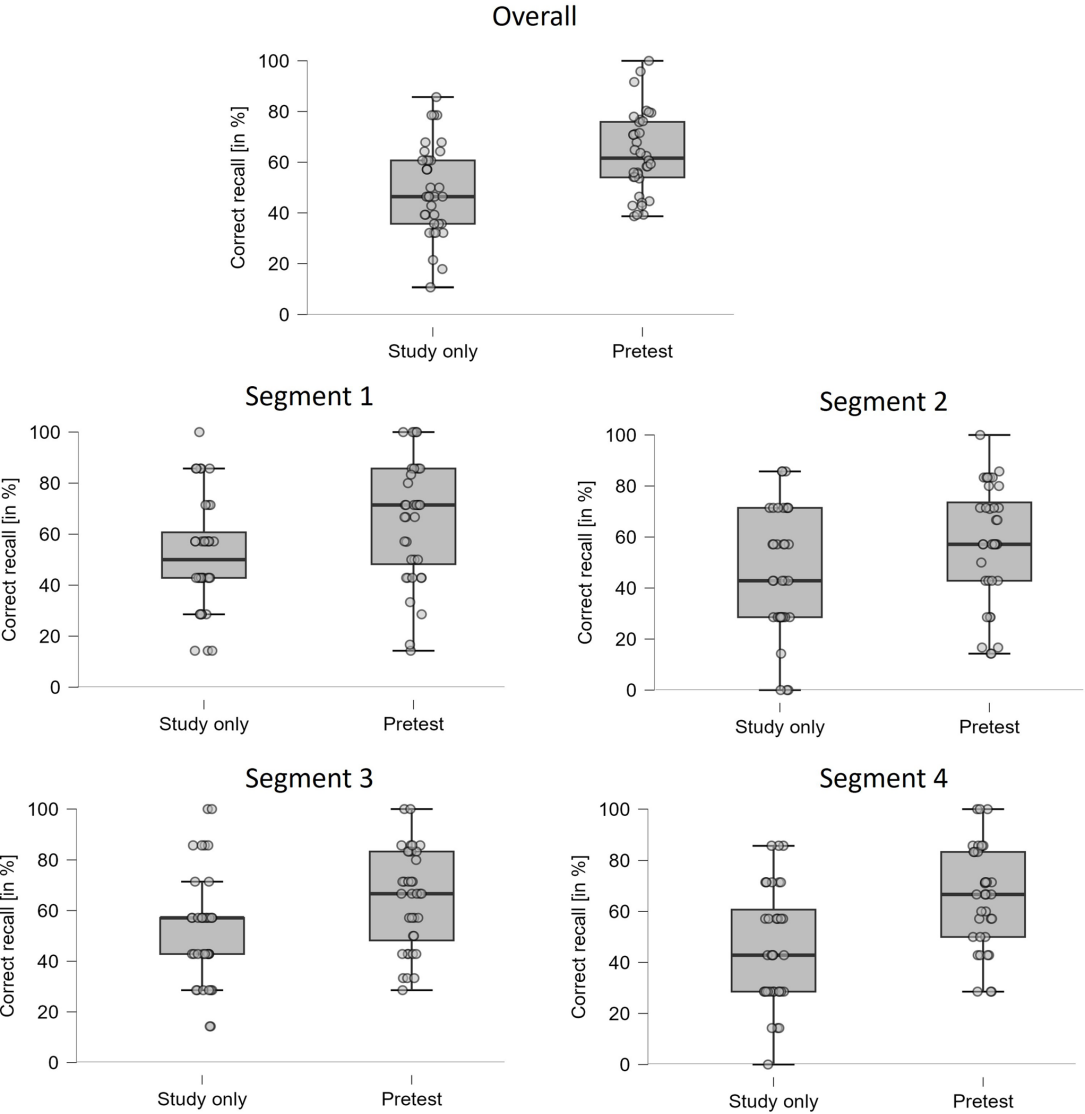
Results of Experiment 2. Box plots with jittered data points depicting recall performance on the final test (in %) as a function of TYPE OF PRACTICE (study only vs. pretest). Each data point reflects recall performance of a single participants. The box plot at the top of the figure shows recall performance averaged across all four segments. The box plots below show mean recall performance separately for the four text segments

Figure 2 shows the percentage of correctly answered questions on the final test as a function of the between-subjects factor TYPE OF PRACTICE (study only vs. pretest) and the within-subjects factor STUDY SEGMENT (segment 1 vs. segment 2 vs. segment 3 vs. segment 4). A 2 × 4 ANOVA of the two factors on correct-recall performance revealed a significant main effect of type of practice, *F*(1,70) = 12.14, *MSE* = 1156.27, *p* < .001, *η*_*p*_^2^ = 0.15, reflecting a reliable pretesting effect with an overall greater recall performance of pretested material relative to material that was studied only (63.4% vs. 49.4%), but no significant main effect of STUDY SEGMENT, *F*(3,210) = 2.43, *MSE* = 275.19, *p* = .07, *η*_*p*_^2^ = 0.03. Most important, there was no significant interaction between the two factors, *F*(3,210) = 1.03, *MSE* = 275.19, *p* = .38, *η*_*p*_^2^ = 0.02, suggesting that the magnitude of the pretesting effect did not vary reliably across segments.

#### Final test – intrusions

Table 1 shows the number of intrusions produced on the final test as a function of the factors TYPE OF PRACTICE and STUDY SEGMENT. A 2 × 4 ANOVA of the two factors on number of intrusions revealed no significant main effects of TYPE OF PRACTICE, *F*(1,70) = 1.09, *MSE* = 3.91, *p* = .30, *η*_*p*_^2^ = 0.02, and study segment, *F*3,210) *<* 1, and also no interaction between factors, *F*(3,210) *<* 1.

## Discussion

The results of the present two experiments demonstrate that pretesting interspersed between the study of multiple segments can boost recall on a delayed final test, both when the segments to be studied are related (Experiment 1) and when they are distinct (Experiment 2). Experiment 1 provided a conceptual replication of the findings reported in Pan et al. (2020) by demonstrating that, for related study segments, a (relatively difficult) free-answer format used during initial pretesting can still enhance later retention of the segments. Indeed, while participants correctly answered only about 2% of the pretest questions in our Experiment 1, in the Pan et al. study, they correctly answered about 50% of the pretest questions. The fact that the current Experiment 1 was able to replicate the earlier study is also noteworthy since, unlike in the earlier study, (i) all questions for which correct answers were produced during pretesting were removed from further analyses and (ii) participants in our study were given less time to study the segments in the pretest than study-only condition (2 min 20 s vs. 3 min 30 s) to equalize the duration of the initial acquisition phase for the study-only and pretest conditions. In Experiment 2, we again employed pretest questions without answer options – of which about 6% were completed correctly – and excluded all questions for which correct answers were given during the initial pretest from further analyses. Results showed that interpolated pretesting can still benefit subsequent recall of the study segments when each segment contains a distinct topic, thus extending the findings of the present Experiment 1 and the Pan et al. study.

Crucially, the results of the present Experiments 1 and 2 did not yield any evidence that the magnitude of the pretesting effect decreased from earlier to later study segments. Since the percentage of correct answers during initial pretesting was very low, one might assume that participants’ engagement with the learning task diminishes from the study of earlier to later segments in the pretest condition, which could lead to a reduced pretesting effect. Rather, the lack of a PRACTICE FORMAT × STUDY SEGMENT interaction on final-test recall in either of the two present experiments suggests that the pretesting effect was quite stable across segments, with the size of the effect even showing a slight numerical increase for later segments. Indeed, when pooling final-test recall performance for the two earlier study segments (segments 1 and 2) and the two later segments (segments 3 and 4), the magnitude of the pretesting effect was 11.9% for the two earlier segments versus 15.2% for the two later segments in Experiment 1, and it was 13.0% for the two earlier segments versus 14.7% for the two later segments in Experiment 2. The present findings thus suggest that even a very low percentage of correct answers during interpolated pretesting does not prevent a continuous pretesting effect in a multiple-segment learning task.

While most studies examining the pretesting effect did not report intrusion errors, the few studies that did typically showed that pretesting leads at least to a numerical reduction in number of intrusions (e.g., Grimaldi & Karpicke, 2012; Kliegl et al., 2024a). The findings of the present two experiments appear not to align with the prior work, since they suggest that the number of intrusions was relatively similar for both the study-only and pretest conditions. Future research may want to investigate whether interpolated pretesting can both enhance correct recall *and* reduce intrusions when the time to study the text segments is equalized for the study-only and pretest conditions.

### Theoretical aspects

The current findings may have implications for the potential processes underlying the pretesting effect, which have often been suggested to be elaboration and/or attention processes. Elaboration accounts of the pretesting effect assume that initial pretesting can boost the activation of information related to a question or retrieval cue, which, on the final test, facilitates access to the correct response (e.g., Richland et al., 2009). In contrast, the core assumption of attention accounts is that a pretest format leads to increased attention to the correct answer feedback presented afterwards, resulting in better recall of the correct response on the final test (e.g., Potts & Shanks, 2014). Elaboration and the attention accounts do not contradict each other since, for instance, pretests may trigger both elaboration processes and increases in attention directed towards the target information once it is presented during feedback. Recent research indeed has yielded evidence that both types of processes may play a central role for the pretesting effect (e.g., Bartl et al., 2024; Huelser & Metcalfe, 2012; Potts et al., 2019; Sana & Carpenter, 2023). The present study was not designed to test whether elaboration or attention processes are the main cause of the benefit of interpolated pretesting. However, the observation that the magnitude of the pretesting effect did not decrease from earlier to later segments in both experiments seems to reveal some important information about the nature of the underlying processes, suggesting that they can be effectively triggered even after multiple rounds of pretesting. It should be a high priority for future work to examine whether elaboration or attention processes primarily underlie the interpolated pretesting effect, and whether and how both processes interact to induce the effect.

Certainly, there may be other factors besides elaboration and/or attention processes contributing to the benefits of interpolated pretesting. For instance, it is possible that because the final test questions already appear on the pretest, the exposure to the target content later covered in the final test may have made it easier to recall the correct final test answers in the pretest than study-only condition. A future study could test this possibility by increasing the similarity between the two conditions and presenting participants in the study-only condition with simple statements of the target content before they study the text passage. Consequently, the level of prior exposure to the target content would be (mostly) the same for the pretest and study-only conditions. 1

Another potential factor contributing to the current interpolated pretesting effect is that participants in the pretest condition may have been more inclined to review the study material or look up answers during the 24-hour delay than participants in the study-only condition. While we are aware of this possibility, we deemed it important from an applied perspective to use a relatively long retention interval since information often needs to be retained over long periods of time in real-life learning scenarios. In addition, based on our experience with learning experiments applying longer retention intervals, participants do not appear to be highly motivated to practice the study material during the delay. Indeed, anecdotal evidence from prior studies in which we asked participants at the end of the experiment whether they actively reviewed any of the study material during the delay suggests that such practice almost never happens.

### Relation to prior work

Earlier research on test-enhanced learning has shown that not only pretests, but also practice tests conducted after study of material to be learned (i.e., posttests) can enhance later retention of the material, the so-called testing effect (e.g., Roediger & Karpicke, 2006; for a review, see Karpicke, 2017). When such posttests are interpolated between the study of multiple item lists, they can promote later recall performance on a cumulative test on all initially studied material (Szpunar et al., 2007, 2013). The finding from the present study – together with the Pan et al. (2020) study – that interpolated pretesting can enhance later recall performance on a cumulative test on all initially studied material thus suggests a parallel between the effects of posttesting and pretesting on memory, adding to some further parallels between the two types of testing situations uncovered in prior research. Indeed, both posttesting and pretesting have been found to reduce forgetting over time (Roediger & Karpicke, 2006; Kliegl et al., 2024a), diminish detrimental effects of competing information at the time of final testing (Halamish & Bjork, 2011; Kliegl et al., 2023), and increase in size with the number of initial test cycles (Karpicke & Roediger, 2007; Kliegl et al., 2024b).

Unlike the present study, Pan et al. (2020) not only showed that interpolated pretesting can promote later retention of pretested material, but that the benefits of interpolated pretesting can also improve participants’ answers to new final-test questions that required participants to recall information from the study segments that was initially not pretested. While these findings generally align with several prior studies showing that pretests can sometimes foster transfer to previously studied but untested information (e.g., Little & Bjork, 2016; Pan & Sana, 2021), a number of studies has failed to find any such transfer effects (e.g.Kliegl et al., 2024a; James & Storm, 2019; Richland et al., 2009). A priori, it thus remains unclear whether interpolated pretesting might generally induce transfer. Critically, however, Sana and Carpenter (2023) recently showed that pretest-induced transfer effects can be boosted when the pretested target material appears in the later part of the subsequently studied prose passage, but not when it appeared in the earlier part of the prose passage. The researchers explained this pattern of results by suggesting that pretesting might open an attentional window that benefits encoding of all information, including non-pretested information, that is encountered before the pretested information is identified in a prose passage. A future study therefore might examine whether interpolated pretesting might induce transfer effects particularly when the pretested information appears later in the prose passage.

*To conclude*, the results of the present study show that interspersing pretests between the study of multiple text segments can promote recall performance of the segments on a later final test, regardless of whether the segments were thematically related or distinct. Since in prior work on the pretesting effect, researchers have primarily used single-“list” tasks, the current findings provide a critical generalization by showing that the benefits of pretesting extend also to multiple-lists tasks, which suggests that this type of pretesting could play a critical role in educational practice.

## Data Availability

All study materials and data have been made publicly available on the Open Science Framework and can be found at https://osf.io/amzct/.

## Footnotes

1 We thank an anonymous reviewer for this suggestion.
